# The Role of the Thyroid Axis in Fish

**DOI:** 10.3389/fendo.2020.596585

**Published:** 2020-11-06

**Authors:** Cole K. Deal, Helene Volkoff

**Affiliations:** Departments of Biology and Biochemistry, Memorial University of Newfoundland, St. John’s, NL, Canada

**Keywords:** fish, thyroid, hormones, reproduction, osmoregulation, feeding, metabolism

## Abstract

In all vertebrates, the thyroid axis is an endocrine feedback system that affects growth, differentiation, and reproduction, by sensing and translating central and peripheral signals to maintain homeostasis and a proper thyroidal set-point. Fish, the most diverse group of vertebrates, rely on this system for somatic growth, metamorphosis, reproductive events, and the ability to tolerate changing environments. The vast majority of the research on the thyroid axis pertains to mammals, in particular rodents, and although some progress has been made to understand the role of this endocrine axis in non-mammalian vertebrates, including amphibians and teleost fish, major gaps in our knowledge remain regarding other groups, such as elasmobranchs and cyclostomes. In this review, we discuss the roles of the thyroid axis in fish and its contributions to growth and development, metamorphosis, reproduction, osmoregulation, as well as feeding and nutrient metabolism. We also discuss how thyroid hormones have been/can be used in aquaculture, and potential threats to the thyroid system in this regard.

## Introduction

The thyroid gland is a key metabolic regulator in the body of animals. An intact axis between the brain, thyroid, and peripheral tissues is essential to modulate energy expenditure and homeostasis (1). An imbalance in energy homeostasis results in the release of brain or peripheral signals, which communicate to the thyroid to increase or decrease energy expenditure, by modulating the release of thyroid hormones (THs). In mammals, there is clear evidence that increased TH production/release induces increases in metabolic rate (2), weight loss (3), and cardiac output (4), while decreased TH production/release leads to opposite effects. In all vertebrates, THs are key hormones that influence a number of physiological processes including growth, development/morphogenesis, and metabolism (5). However, in fish, the role of the thyroid is incompletely understood. Although homology in genetic mechanisms exists between mammals and fish (6) and THs are generally conserved in structure and function (7), the thyroid system is not always analogous between groups.

Fish [Chondrichthyes (i.e., cartilaginous fish: sharks, skates, rays), Osteichthyes (i.e., bony fish: ray-finned and lobe-finned fish) and Agnatha (i.e., jawless fish: hagfish and lamprey)] (8) make up approximately 48% of all vertebrates (9), contributing to the 73,327 of total vertebrate species described (10). This diversity has led to wide variations within ecological niches, physiological mechanisms and local adaptations. In the context of the thyroid, major differences in terms of morphology, physiology, and regulation are seen within and between species.

The thyroid was first described in fish in the 19^th^ century (11). Later studies compared the structure/location of the gland in different fish species [e.g., gill tissue in rainbow trout (*Oncorhynchus mykiss*) (12)], and uncovered the role of the thyroid as a regulator of metabolic activity (13), and the role of the pituitary [sailfin molly (*Poecilia latipinna*) (14)] and hypothalamus [African lungfish (*Protopterus annectens*) (15)] in the regulation of thyroid function. Despite over a century of research, our knowledge of the physiology of the fish thyroid is still incomplete, and previously published reviews focus on teleosts and on specific functions of the thyroid [e.g., metamorphosis (16); reproduction (17)].

This review provides a general overview of our current knowledge on the actions of thyroid hormones in fish (not only teleosts but also other groups), including those on growth and development, reproduction, osmoregulation, and feeding/metabolism, how thyroid function may be affected by intrinsic and extrinsic factors, and how this knowledge could be used by the aquaculture industry.

## Thyroid Hormones and the Thyroid Axis

### Regulation of Secretion

THs consist of two forms, thyroxine (or tetraiodothyronine, T_4_) and the biologically active triiodothyronine (T_3_) (18). Although T_4_ is the predominant circulating form, T_3_ is more biologically active (19). Conversion of T_4_ to T_3_ occurs in central and peripheral tissues (e.g., brain, gut, liver) by enzymatic removal (5’-monodeiodination, 5’-MDA) of an iodide unit on the outer ring of T_4_ (20).

In vertebrates, the secretion of THs is regulated by the hypothalamus-pituitary-thyroid (HPT) axis (hereafter also referred to as the thyroid axis). The prime stimulatory hormone for the thyroid gland/follicle is thyrotropin (TSH), from thyrotropes of the anterior pituitary. In higher vertebrates, thyrotropin-releasing hormone (TRH) is the main stimulator of TSH release, whereas some neurotransmitters, dopamine (DA), and somatostatin (SS), act as inhibitors (21, 22). Serum TH levels have direct inhibitory effects on the synthesis and release of both hypothalamic TRH and pituitary TSH (23). While it is clear in mammals that TRH stimulates release of TSH from the anterior pituitary, the role of TRH in activating the fish thyroid axis is not clear (17).

In teleosts, there seems to be species-specific differences in TRH action on thyrotropes. In bighead carp (*Aristichthys nobilis*), TRH treatment of pituitary cells increases TSHβ messenger RNA (mRNA) expression levels (24). However, in common carp (*Cyprinus carpio*) (25, 26) and coho salmon (*Oncorhynchus kisutch*) (27), TRH does not directly affect TSH expression or release from the pituitary. It has been suggested that, in some teleosts, corticotropin-releasing hormone (CRH) may play a greater role as a TSH stimulator than TRH (27, 28).

There is evidence that TRH stimulates the secretion of growth hormone (GH), prolactin (PRL), adrenocorticotropic hormone (ACTH), and melanocyte stimulating hormone alpha (α-MSH) in fish (29). TRH evokes release of proopiomelanocortin (POMC)-derived peptides (α-MSH and ACTH) (30) and GH (31) from goldfish (*Carassius auratus*) anterior pituitaries, and PRL synthesis and release in common carp (25). It is possible that TRH-induced increases in T_4_ plasma levels, as seen in rainbow trout and Arctic charr (*Salvelinus alpinus*) (32), might occur through stimulation of TSH release or other pituitary hormones such as GH and PRL.

Similar to mammalian TSH, fish TSH is a glycoprotein that comprises a hormone-specific β subunit (TSHβ) coupled to a glycoprotein α subunit (GSUα) [e.g., teleosts (33), elasmobranchs (34)]. The α subunit is common to TSH and gonadotropins [luteinizing hormone (LH) and follicle-stimulating hormone (FSH)] whereas the β subunit confers hormonal specificity (34). TSH mRNA is mainly expressed in teleost pituitary tissue, although ectopic expression occurs, particularly in gonads (33).

TSH exerts its actions by binding to TSH receptors (G protein-coupled receptors) on the basal membrane of thyroid follicles (33). Two TSH receptor sequences have been identified in most teleost groups but only one receptor gene has been identified in the coelacanth and elephant shark genomes (34). Evidence suggests that, in fish, TSH has a stimulatory effect on the synthesis/release of THs and iodide uptake. For example, incubating thyroid glands from the sea catfish (*Galeichthys felis*) *in vitro* for 3 days with mammalian TSH increases T_4_ release and thyrocyte height (35); *in vivo* injections with mammalian TSH increase thyrocyte height and follicle proliferation in coho salmon (36), and circulating T_4_ levels in mummichog (*Fundulus heteroclitus*) (37) and brook trout (*Salvelinus fontinalis*) (38).

The release of pituitary TSH is inhibited by DA (39) and SS (40), neuropeptides, and by negative feedback actions by T_4_ and T_3_. In goldfish, treatment with SS suppresses radioiodide uptake by thyroid follicles but does not lower plasma T_4_ in TSH-injected goldfish, supporting the role of SS as a TSH inhibiting factor in this species (41). Appetite regulating peptides also affect TSH expression/release at the pituitary, as leptin and β-endorphin stimulate, whereas galanin and neuropeptide Y (NPY) inhibit TSH pituitary mRNA expression in bighead carp (42).

In mammals, THs exert an inhibitory feedback action on TRH and TSH expression by binding to TRβ located on the TRH promoter in the hypothalamus (43, 44), and inhibiting the transcription of both TSHα and TSHβ in the pituitary (45). In fish, there is no clear evidence of TH inhibition on TRH. Injections of T_4_ in common carp have no effect on hypothalamic TRH expression, but increase hypothalamic CRH binding protein expression (46), which might result in CRH inactivation and in the modulation of TSH synthesis in the pituitary, as seen in mammals (47). There is however evidence in fish for feedback control of THs at the pituitary level, as THs decrease pituitary TSHβ expression both *in vivo* [e.g., goldfish (48); turbot (*Scophthalmus maximus*) (49); European eel (*Anguilla Anguilla*) (50)] and *in vitro* [goldfish (51)].

### Thyroid Hormone Synthesis Sites and Peripheral Regulation

Synthesis of THs occur in thyroid follicles—a single layer of epithelial cells (thyrocytes) enclosing a colloid-filled space (52). In mammals, and most vertebrates, the thyroid gland is an encapsulated gland in the neck region. In fish, the thyroid gland can be either compact/encapsulated [e.g., Chondrichthyes or cartilaginous fish, such as sharks and rays, and Chondrostei, such as sturgeons] or more commonly diffusely arranged in the pharyngeal, heart, and kidney regions [e.g., most teleosts with a few exceptions such as Tetraodontiformes and Lophiiformes] (53–55). In larval lampreys, the site of TH synthesis is the subpharyngeal endostyle, a filter-feeding apparatus, which transforms into typical follicular thyroid tissue during metamorphosis (56).

Synthesis of THs requires iodine, that, in most fish, is assimilated by diet or from water *via* the gills (57), and thyroid uptake of iodine requires TSH binding to follicles. Evidence on TSH stimulation of iodide uptake in teleost fish is scarce as the spatial distribution of thyroid follicles makes it difficult to measure radioiodide uptake (38), but it has been shown in elasmobranchs, who have an encapsulated thyroid [e.g., lesser spotted dogfish (*Scyliorhinus canicula*) (58)].

Once secreted from follicles, THs require peripheral regulation to exert their effects. Iodothyronine deiodinases are selenoenzymes that regulate TH availability and disposal. Several isoforms of deiodinases (DIOs) with different catalytic properties (type 1, 2, and 3, or DIO1, DIO2, DIO3) and tissue- and developmental stage-specific expressions exist (59). In mammals, DIO2 is part of the activating pathway [or outer ring-deiodination (ORD)] as it converts T_4_ to T_3_, whereas DIO3 is part of inactivation [inner ring-deiodination (IRD)] as it converts T_4_ and T_3_ to inactive metabolites [reverse triiodothyronine (rT_3_) and 3,3^′^-diiodothyronine (T_2_)] (59, 60). DIO1 is capable of both activation (ORD) and inactivation (IRD), processing T_4_ to T_3_ and rT_3_ to T_2_, respectively (61, 62). Similar DIOs have been shown in fish (57, 63–66). However, fish DIOs differ in some respects from their mammalian counterparts (20). For example, teleostean DIO1 is resistant to propylthiouracil (PTU, inhibitor of thyroperoxidase, TPO—responsible for iodide to iodine oxidation in thyroid follicles) inhibition, and teleosts have relatively higher levels of hepatic DIO2 activity and expression compared to other vertebrates (67).

### Regulation by Circadian and Seasonal Rhythms

Several studies have shown circadian and seasonal cycles of THs and thyroid axis components. In mammals, circadian cycles of TRH and TSH are controlled by “pacemakers” within the superchiasmatic nucleus (SCN) of the hypothalamus. These in turn regulate circulating TH levels (68). The pineal gland—which produces melatonin, and controls sleep patterns in a circadian and seasonal manner—also has an inhibitory influence on circulating THs (69). Studies in hamsters show that melatonin inhibits the release of TSH and increases DIO3 expression during winter months (short photoperiod), and stimulates TSH release in summer (long photoperiods), increases DIO2 expression and decreases DIO3 expression, thus controlling the availability and metabolism of THs (70, 71).

Several studies in fish have shown that thyroid axis components respond to environmental cues (72) and undergo circadian and seasonal cycles (73). Pituitary transcript expression levels of TSH and DIO exhibit distinct rhythms. In red drum (*Sciaenops ocellatus*), seasonal rhythms of T_4_ correlate with pituitary TSH subunits (TSHα, TSHβ) and DIO3 gene expression cycles (74), and in Arctic charr, hypothalamic DIO2 expression is decreased during late summer (75). In fish, there is evidence that the saccus vasculosus (SV, an organ only observed in fish, situated on the ventral side of the diencephalon, posterior to the pituitary gland) is the seasonal sensor in the brain. The SV expresses TSH and DIO2, suggesting that this organ might play a central role in seasonal changes in THs, albeit probably linked to reproduction (76). In precocious male masu salmon (*Oncorhynchus masou*), the SV responds to changes in light, with salmon kept under long periods of light displaying high TSHβ and DIO2 protein levels, the opposite occurring with exposure to short periods of light (77).

TH circadian cycles have been shown in several fish species [see (73)], including Atlantic salmon (*Salmo salar*) (78), winter flounder (*Pseudopleuronectes americanus*) (79), goldfish (80), and red drum (81), although the time of the peak of TH appears to be species-specific. There also appears to be sex-specific TH rhythms, as in rainbow trout, TH levels increase during the day and decrease at night in males, and increase at night and decrease in the morning in females (82). Seasonal variations in THs also exist, often related to migration and reproduction [e.g., channel catfish (*Ictalurus punctatus*) (83); Atlantic cod (*Gadus morhua*) (84); rainbow trout (85)].

## Mechanism of Action and General Actions of Thyroid Hormones

The ability of THs to exert their many pleiotropic effects relies on efficient transport, bioactivation, and genomic/nongenomic actions at target tissues.

### Thyroid Hormone Transport

In higher vertebrates, THs are transported by plasma TH-binding proteins: thyroxine-binding globulin (TBG), transthyretin (TTR), and albumin. The primary plasma TH-binding molecules in fish consist of albumin and prealbumin, the latter now identified as TTR (86). A TBG-like protein has not yet been identified in fish. In contrast to mammals, fish TTR binds T_3_ more avidly than T_4_ (57), possibly making albumin the main T_4_ binding protein (86).

Due to the lipophilic nature of THs, it was previously assumed that passive diffusion across lipid bilayers of plasma membranes occurred. It is now believed that THs enter target cells *via* facilitated transport by several ATP-dependent transporters including the monocarboxylate transporters (MCTs) such as MCT8, organic anion transporter polypeptides (OATPs, predominately present in brain capillaries), large neutral amino acid transporters (LATs), and the sodium/taurocholate co-transporting polypeptide (SLC10A1, also known as NTCP) (87, 88).

With the exception of some studies on the role of MCT8 in zebrafish (*Danio rerio*) development, little is known about TH transporters in fish. The tissue distribution of TH transporters appears to vary between fish models. MCT8 mRNA is expressed in brain, spinal cord and vascular system in zebrafish (89) and mostly in the liver of fathead minnow (*Pimephales promelas*) (90). OATP1C1 is expressed primarily in the liver and brain in zebrafish (91, 92), and in the gonad, liver, and brain in fathead minnow (90).

The expression of TH transporter transcripts shows an inverse relationship to circulating TH levels. In fathead minnow, exogenous T_3_ administration leads to a reduction in liver OATP1C1 transcript abundance (90), while treatment with oral PTU increases brain MCT8 expression (93). In zebrafish, MCT8 seems to mediate T_3_ transport across the blood brain barrier (BBB) (89) and MCT8-deficient zebrafish have altered nervous system development (94). The role of OATPs in fish remains unclear but in zebrafish, OATP1C1 deficiency leads to hyperactivity of the thyroid and the development of goiter (thyroid follicle enlargement), possibly as a consequence of low TH levels as a result of reduced transport into target cells (91).

### Thyroid Hormone Nuclear Receptors

THs affect physiological processes by regulating expression of genes in target tissues (genomic actions) (95). Within target cells, T_3_ binds to thyroid hormone receptors (TRs). TRs are located on thyroid response elements (TRE) of the DNA, located at T_3_ target gene promoter sites (96). Nuclear TRs act as ligand-modulated transcription factors, In the absence of T_3_, TR represses transcription by recruiting corepressors [e.g., nuclear-receptor co-repressor (NCoR)/silencing-mediator for retinoid/thyroid hormone receptors (SMRT)], whereas in the presence of T3, TRs recruit coactivators [e.g., steroid receptor coactivator (SRC), p300/CREB-binding protein (CBP)] to facilitate transcription (96). Therefore, the transcription rate of target genes depends on the binding of T_3_ to TRs.

TRs are products of two different genes, c-erbAα and c-erbAβ (or TRα and TRβ) (97, 98). The TR binds to a TRE as a monomer, a homodimer *(*α/α, α/*β*, *β*/*β*) or a heterodimer, in which a TR isoform dimerizes with the retinoid X receptor (RXR) (99). TRα and TRβ each have different isoforms that have different tissue distributions (e.g., in mice, TRα1 and TRβ1 are expressed in all tissues, but TRα1 is predominantly expressed in the heart and brain, whereas TRβ1 is predominant in skeletal muscle, kidney, and liver) and binding capacities (TRα2 and TRα3 isoforms are truncated and are unable to bind T_3_) (98).

In fish, several species-dependent TR isoforms have been identified. For example, Japanese flounder (*Paralichthys olivaceus*), Atlantic salmon, and Atlantic halibut (*Hippoglossus hippoglossus*) have two distinct TRα genes, while conger eels (*Conger myriaster*) have two subtypes of each TRα and TRβ genes (100–102). Goldfish have three unique TRα isoforms (TRα-1, TRα-2, and TRα-truncated) all similarly expressed in pituitary, brain, liver, gonads, and gut (103). The goldfish truncated form may inhibit transcription of functional TRs by competition for TREs (103, 104). In tilapia, two isoforms of TRβ exist—a short (S-TRβ1) and long (L-TRβ1) isoform—differing by nine amino acids. T_3_ and T_2_ bind to activate L-TRβ1, but not S-TRβ1, and regulate TRβ expression *in vivo* (105).

Differences in the number/type/specificity of isoforms, and tissues distributions might indicate species-specific differential splicing, target cells, and functions, although it must be noted that transcript expression levels might not reflect protein levels, for which information is lacking (95).

### Non-Nuclear Thyroid Hormone Receptors

THs have the ability to act both non-genomically and extracellularly—within the cytoplasm or plasma membrane—in a very rapid manner. THs activate intracellular pathways and other transcription factors such as the mitogen-activated protein kinase (MAPK) (106, 107) or phosphatidylinositol 3-kinase (PI3K) pathways (108, 109) by binding to the integrin α_V_β_3_ TH specific plasma membrane receptor (110). Non-genomic actions may have downstream long-term specific nuclear effects (cell proliferation, gene transcription) leading to cross-talk between non-genomic and genomic action of THs (111).

There is very limited evidence showing direct non-genomic actions of THs in fish, as non-genomic and genomic effects can overlap in the nucleus. In embryonic zebrafish, T_4_, but not T_3_, regulates sodium currents through the MAPK pathway requiring the integrin α_V_β_3_ receptor (112). It has also been suggested that, in fish, THs regulate mitochondrial respiration (113), similar to what is seen in rodents, for which TH binding sites have been shown in mitochondrial membranes (114).

### Actions of T2

Although most studies focus on the actions of T_4_ and T_3_, recent evidence shows that T_2_, a product of T_3_ ORD, is also biologically active and binds to TRβ in teleosts (105). In rodents, administration of T_2_ increases metabolic rate and has hypolipidemic effects (115). In fish, T_2_ regulates the transcription of genes associated with cell signalling and transcriptional pathways in the liver of Nile tilapia (*Oreochromis niloticus*) (116) and stimulates mitochondrial respiration of liver and muscle in goldfish (117). T_2_ (like T_4_ and T_3_) also decreases DIO1 and DIO2 activities in the liver of killifish (*Fundulus heteroclitus*) (66), and regulates thermal acclimation in zebrafish (118) and growth in tilapia (119). Therefore, while previously viewed as an inactive TH, T_2_ may have a larger role than originally thought.

## Role of the Thyroid Axis on Somatic Development and Growth

In fish, as in all vertebrates, THs are crucial for the proper development of both embryos and adults, and are involved in major life transitions and metamorphosis in some species (52, 120, 121).

### Maternal Origin of Thyroid Hormones and Importance in Egg and Larval Development

In early mammalian development, an embryo relies solely on maternal THs as its thyroid gland is not yet fully functional (121). THs are actively transported from the mother to the embryo across tissue barriers—including the placenta and BBB—and act on embryonic target cells (121).

The diverse modes of reproduction in fish (122) result in species-specific thyroid-mediated development, due to the variety of mechanisms by which maternal transfer of THs into the egg/embryo occurs (123).

Most fish have external fertilization and are oviparous [i.e., produce eggs that develop and hatch in the external environment (124)]. Others have internal fertilization and the egg/embryo develops within the mother. In viviparity, eggs develop and hatch within the mother before being released as live young to the external environment (124). In yolk sac, or lecithotrophic viviparity, eggs are retained inside the female until fully developed, with no maternal chemical contribution beyond yolk. In matrotrophic viviparity, the embryos receive additional nutrition from the mother (e.g., maternal proteins and lipid-rich histotroph secreted from the uterus in histotrophy; unfertilized eggs/other embryos in oophagy/adelphophagy; or through placenta-like structures) (125, 126).

In oviparous fish, there is evidence that THs are transferred from female fish to eggs (127). Fathead minnow and zebrafish eggs display high TH levels and high transcript levels of thyroid-related transcripts (TRα, TRβ, DIO1, DIO2, DIO3, TPO, sodium-iodide symporter, TRH-receptor, TSH-receptor, TG, and TTR) before 2–3 days post-fertilization (dpf)—time at which endogenous TH production begins—suggesting a maternal transfer of THs (123). In alligator gar (*Atractosteus spatula*) and spotted gar (*Lepisosteus oculatus*), injecting females with THs or TSH results in increases in the concentrations of T_4_ and T_3_ in early embryos (128). As well, maternal injections and egg immersion have been shown to increase pigment concentrations in larval tissues, hatching and larval growth rate, swim bladder inflation, muscle development, larval metabolic capacity, and metamorphosis [e.g., Sterlet sturgeon (*Acipenser ruthenus*) (129, 130); piracanjuba (*Brycon orbignyanus*) (131); matrinxã (*Brycon amazonicus*) (132); zebrafish (133); goldfish (134)]. Interestingly, it appears that T_4_ concentrations are greater than T_3_ concentrations in eggs of most freshwater (FW) fish, whereas T_3_ concentrations are greater in seawater (SW) fish (135), suggesting differential TH utilization during egg development.

Less is known about maternal transfer of THs in viviparous species. In the lecithotrophic viviparous dogfish (*Squalus acanthias*), 5′-MDA activity (an indicator of the production rate of the active thyroid hormone T_3_) is present in yolk sac embryos and may be of maternal origin (136), and in Korean rockfish (*Sebastes schlegelii*), maternal T_3_ injections improve growth and survival of young *in utero* (137). In matrotrophic viviparity, there is an association between embryos and maternal structures, suggesting that maternal THs could be exchanged (125). In surfperch (*Neoditrema ransonnetii*)—a matrotrophic teleost in which embryos are sustained by ovarian cavity fluid (OCF) ingestion and by nutrient absorption *via* enlarged hindgut—OCF and fetal plasma contain high TTR levels. TTR plasma levels are higher in pregnant fish than in non-pregnant fish, and large amounts of maternal TTR are taken up by fetal intestinal epithelial cells (enterocytes), indicating that maternal TTR is secreted into OCF and taken up by fetal enterocytes, presumably to deliver THs to developing embryos (138). In the viviparous bonnethead shark (*Sphyrna tiburo)*, yolk-dependent embryos undergo yolk-sac modification in which the fetal portion of a placenta attaches to the maternal uterine wall near mid-gestation, which facilitates direct exchanges of blood and nutrients between the mother and embryo (139). In this species, T_3_ in yolk increases from pre- to post-ovulation and peaks during the pregnancy stage, and maternal serum T_3_ concentrations increase as development progresses, suggesting that maternal THs are needed for development of the egg/embryo (140).

### The Thyroid and Growth Axes

In fish, as in mammals, somatic growth is regulated by hormones of the growth (or hypothalamic–pituitary–somatotropic, HPS) axis, i.e., growth-hormone releasing hormone (GHRH) from the hypothalamus, and growth hormone (GH) produced by somatotrophs in the anterior pituitary. GH release is stimulated by GHRH and other secretagogues (e.g., ghrelin) and inhibited by SS (141). GH has direct and indirect actions on tissues *via* the stimulation and release of insulin-like growth factors I and II (IGF-I, IGF-II) by the liver. These act on tissues to promote cellular proliferation and differentiation (142, 143).

Embryonic differentiation/organogenesis and growth in teleosts is regulated by THs, likely by triggering both GH [e.g., THs increase GH mRNA transcription in rainbow trout (144) and carp (145), and increase synthesis and release in hybrid tilapia (146)] and IGF-I [e.g., THs induce *in vivo* and *in vitro* synthesis/release in Mozambique tilapia (*Oreochromis mossambicus*) (147)]. Since THs are crucial regulators of growth (148, 149), inhibition of thyroid function results in impairment in the development of brain, skeleton, and other organs, as well as in pigmentation. For example, in zebrafish, treatment with T_3_ increases IGF-1 expression and enhances swim bladder and eye development but IGF-1 receptor blockade suppresses these effects of T_3_ on swim bladder and eye (150).

#### Interactions Between Thyroid and Growth Axes

Components of the thyroid axis have been shown to affect the GH/IGF-I axis in vertebrates. TRH stimulates the secretion of GH by acting directly upon GH cells in amphibians (151, 152) and reptiles (152, 153). In rodents, THs have been shown to stimulate GH synthesis and secretion (154, 155), upregulate SS receptors (156) and increase SS immunoreactivity and release (157).

In fish, the effects of the thyroid axis on growth are not clear, as components have been shown to have both inhibitory and stimulatory effects. TRH increases GH secretion *in vivo* in goldfish (158) and tilapia hybrid (*Oreochromis niloticus x Oreochromis aureus*) (146), and *in vitro* in common carp pituitary fragments (159), but not in tilapia hybrid (146) or sailfin molly (160). TSH injections increase GH plasma levels in several species including Nile tilapia (146), killifish (161, 162), coho salmon (163), rainbow trout (164), and Indian carp (*Cirrhinus mrigala*) (165).

THs also affect the growth axis in fish, although results are inconsistent. *In vivo* treatment with T_4_ or T_3_ decreases both pituitary and serum GH levels in female European eel (166) but has no effect on GH levels in goldfish (51). T_4_ administration to aquarium water increases somatotroph activity in red belly tilapia (*Coptodon zillii*) (167), and *in vivo* T_3_ injections increase pituitary GH mRNA expression in rainbow trout (144) and GH plasma levels in hybrid tilapia (146). THs also act on liver to stimulate IGF-I synthesis/secretion: T_3_ increases hepatic IGF-I mRNA levels both *in vitro* and *in vivo* in Mozambique tilapia (147) and zebrafish (168), but not in coho salmon (169) or silver sea bream (*Sparus sarba*) (170). T_3_ may regulate IGF-I expression by binding to liver GH receptors [e.g., coho salmon (169)] or TRs [e.g., rainbow trout (171)], although this action seems species-specific.

Whereas the thyroid axis can affect growth, components of the growth axis affect the thyroid. In mammals, the thyroid axis is stimulated by GH, as seen by increases in TH levels following GH treatment (172), and inhibited by SS (173). In humans, ghrelin decreases TSH-induced production of thyroglobulin and mRNA expression of TPO in thyroid cells (174), while SS treatment decreases the volume of TSH-cells and serum concentrations of TSH in rats (175) but has no effect on serum TSH and TH levels in humans (176).

In fish, there is also evidence for a role of the GH axis in regulating thyroid function. TSH receptor expression is up-regulated in transgenic grass carp overexpressing GH (177), and in European eel, GH stimulates thyroid follicles to release T_4_ and enhances peripheral 5’-MDA activity (178). In mummichog, hypophysectomy prevents TSH-induced secretion of T_4_ and treatment with ovine GH restores this response (162). Information on the role of ghrelin and SS on the thyroid axis is scarce. Plasma TH levels are inversely correlated with SS plasma levels in rainbow trout (179), and burbot (*Lota lota*) have decreased plasma ghrelin and TH levels pre-spawning (180), suggesting an interaction between SS, ghrelin and THs.

### Ecological Importance of Thyroid-Mediated Development

THs are particularly important for the development of the central nervous system (CNS) and for ecological/ecosystem shifts within fish. The plasticity of the fish nervous system allows it to regenerate after injury and be remodeled during life history shifts, processes in which THs are most likely implicated. This has been demonstrated in zebrafish submitted to optic nerve injury, in which the re-innervation of the optic tectum is accelerated when T_3_ plasma levels are lowered with a TRβ antagonist and iopanoic acid (IOP, inhibits TH release and reduces peripheral T_4_ to T_3_ conversion) (181).

In the case of migrating anadromous species, T_3_ induces the proliferation of olfactory receptor neurons (which are crucial for natal stream imprinting) in olfactory epithelium (182) and T_4_ induces a switch from UV to blue opsin photoreceptors in the retinas of young coho salmon and rainbow trout (183)—which allows better visual contrast for feeding before a SW migration (184). In masu salmon, T_3_ binding in the brain is tissue-specific during the parr-smolt transformation: At both life stages, T_3_ binding is highest in the olfactory epithelium, and smolts show higher binding compared to parr in this region (185). This suggests that THs play an important role in functional changes of the brain and olfactory epithelium, playing a preparatory role for shifting between aquatic habitats.

## Metamorphosis

Fish metamorphosis refers to the dramatic changes seen in flatfish, lampreys, and eels, but also be applied to any irreversible post-embryonic developmental event that affects multiple physiological or morphological traits (excluding those related to sexual maturation, reproduction, or senescence) seen in several FW and marine species (56, 186). THs are key regulators of teleost metamorphosis, which involves cellular and molecular remodeling that lead to developmental changes (16). Typically, thyroid activity is low during pre-metamorphosis (i.e., low TH levels, with reduced DIO and TR expression), increases during the metamorphic event, peaks during developmental changes (metamorphic climax), and decreases to pre-metamorphic levels (16, 186).

In flatfish, pelagic larvae develop symmetrically with eyes on each side of the head, and morph into asymmetric benthic juveniles following the migration of one eye to the opposite side of the head to become right- or left-eyed, a species-specific distinction [e.g., right-eyed Atlantic halibut (187), left-eyed Japanese flounder (100) and left- or right-eyed Starry flounder (188)]. In Senegalese sole (*Solea senegalensis*), increases in TH circulating levels, pituitary TSHβ, and whole body thyroglobulin and TR transcript levels (189) coincide with metamorphic climax and activity in thyroid follicles (190). Similarly, during Atlantic halibut metamorphosis, the vast majority of transcripts expressed in the head transcriptome are related to the thyroid axis (187).

In sea lamprey (*Petromyzon marinus*), the blind, sedentary, filter-feeding larvae metamorphose into free-swimming juveniles. This involves major changes including the development/transformation of adult kidneys, GIT, gills, and the development of the eyes (56). Interestingly, as opposed to other fish, lamprey metamorphosis coincides with a drop in serum endostyle cells-derived TH levels, is blocked by TH treatment and is stimulated by goitrogens (which suppress TH levels), but the mechanisms by which this occurs are still unclear (56, 191).

In diadromous species, which migrate between SW and FW, metamorphosis induces morphological and physiological changes (e.g., changes in body shape, pigmentation, kidneys, gut, eyes, osmoregulation, metabolism) that prepare the fish to survive in a new habitat (186). In anadromous salmonids (e.g., *Oncorhynchus*, *Salmo* and *Salvelinus*), fish hatch and grow in FW before migrating to SW where most of the somatic growth takes place. Smoltification [or parr (FW fish)–smolt (SW fish) transformation] refers to the changes in physiology, behavior, and morphology that occur in juvenile salmonids prior to this migration. These include pigmentation changes (i.e., body and darkening of fins) and changes in olfactory receptors and osmoregulatory adaptation (192–195), all associated with a surge in TH levels. For example, TH treatment induces downstream migration in Atlantic (196), coho, chum (*Oncorhynchus keta*) and sockeye (*Oncorhynchus nerka*) salmon (197), and TSH injections or TH treatment increase purine synthesis, which is responsible for skin silvering in rainbow trout (198) and brook trout (199).

In contrast to salmonids, eels hatch and develop as marine larvae [flat and transparent marine larvae (leptocephali)] and undergo a SW to FW (catadromous) migration. Larvae transform into transparent “glass eels,” which move to FW and complete metamorphosis to become juvenile “elvers.” These then undergo a secondary metamorphic event (silvering) and return to the ocean for spawning. In Japanese eel, the change from leptocephalus larvae to glass eel is characterized by an increase in TH levels and TSHβ expression, with TSHβ levels peaking at the glass eel stage and THs increasing into the juvenile stages (200).

Many teleosts undergo subtle irreversible post-embryonic morphological and physiological changes that have been defined as a metamorphosis and are regulated in part by THs (186). These include the development of the fins and the appearance of adult stripes in zebrafish (133), and changes in coloration and swimming behavior marine fish such as red sea bream (*Pagrus major*) (201), grouper (*Epinephelus coioides*) (202), surgeonfish (*Acanthurus triostegus*), and clown fish (*Amphiprion ocellaris*) (203).

## Reproduction

THs regulate many aspects of the reproductive system, including formation of gametes and steroids, and sexual behavior in both males and females. In vertebrates, the hypothalamus-pituitary-gonadal (HPG) axis regulates reproduction: gonadotropin releasing hormone (GnRH) from the hypothalamus stimulates the pituitary to release gonadotropins (GTH) [luteinizing hormone (LH) and follicle stimulating hormone (FSH)] which act on gonads to regulate gametogenesis and steroidogenesis [e.g., in mammals (204) and fish (205)]. There is growing evidence of a crosstalk between the thyroid and HPG axes in several vertebrates (e.g., mammals, amphibians, fish) (206).

In mammals, the link between thyroid and reproductive function is well established. THs and TSH can affect gonadal development and sex steroid hormone synthesis and actions, and thyroid dysfunction is associated with decreased fertility, impaired gonadal function and disruption of seasonal cycles in both in males and females (207–210). In fish, the link between THs and reproduction is not clear, as inconsistent results have been reported, likely due to the diversity in reproductive strategies, and methods used to investigate TH actions (211).

### Thyroid Hormone and Reproductive Cycles

Several studies have shown correlations between circulating THs and reproductive cycles (e.g., gamete formation and maturation, and spawning/hatching events) in fish, but between species, the nature of these relationships vary. Among teleosts, some species display peaks in plasma THs during gametogenesis [e.g., rainbow trout (212); brook trout (213) and/or during spawning [e.g., climbing perch (*Anabas testudineus*) (214); sea lamprey (215)], whereas others display decreases in TH levels during gonad maturation [e.g., Mozambique tilapia (216)], before [e.g., sockeye salmon (217)] or during spawning [e.g., winter flounder (79)]. In the jawless Pacific sea lamprey, both males and females show peaks in plasma THs during gametogenesis and spawning (215, 218).

In the Chondrostei stellate sturgeon (*Acipenser stellatus*) and lake sturgeon (*Acipenser fulvescens*), THs are correlated with increased gonad maturation during the spawning season (219, 220), while in immature and previtellogenic individuals, changes in THs during the reproductive season are more closely correlated with temperature, feeding, and growth [e.g., great sturgeon (*Huso huso*) (221) and lake sturgeon (220)].

Very little is known about the role of THs in elasmobranch reproduction. In oviparous elasmobranchs, thyroid activity and TH levels are usually lowest in immature females in the non-breeding season, and greatest during egg development and vitellogenesis during the reproductive season [e.g., lesser spotted dogfish (222); brownbanded bamboo shark (*Chiloscyllium punctatum*) (223)]. Complete thyroid removal inhibits seasonal gonad development [e.g., spotted dogfish (224)]. A similar correlation between thyroidal function and female reproduction has been shown in viviparous elasmobranchs. In the Atlantic stingray (*Dasyatis sabina*), circulating T_3_ levels and thyroid activity are low in immature individuals and high in females undergoing oogenesis, and, from ovulation throughout gestation (225, 226). Similarly, in the torpedo (*Torpedo ocellata*), thyroid activity is high in gestating females (227). However, in female dogfish, thyroid activity does not seem to be associated with reproductive events, but rather with migration (228).

### Evidence of Expression of Deiodinases, Thyroid Hormone Receptors, and Thyrotropin Receptors in Gonads

#### Deiodinases

DIOs have been shown to be present in gonads [e.g., mammals (229, 230); amphibians (231); reptiles (232)] and to be involved in reproductive cyclicity. In mammals, 5’-MDA activity is elevated during gonad development and differentiation [e.g., horse ovary (233); pig testis (230)]. In western clawed frog (*Silurana tropicalis*) gonads, DIO2 and DIO3 expressions increase and DIO1 expression decreases throughout the development into adult (231). Moreover, gender-specific roles of DOIs have been suggested in lower vertebrates. Adult western clawed frog testis show higher expression of DIO1, DIO2, and DIO3 than ovary (231), and in breeding green anole lizards (*Anolis carolinensis*), DIO2 and DIO3 expression levels are high in testes and ovaries, respectively (232).

Although DIO1, DIO2, and DIO3 activity/expression has been shown in the gonads of several fish [including striped parrotfish (*Scarus iseri*) (234), European sea bass (*Dicentrarchus labrax*) (235), goldfish (236), Nile tilapia (237), sapphire devil (*Chrysiptera cyanea*) (238), and rainbow trout (239)] their role in gonadal thyroid metabolism is not clear.

A gender-specific expression of DIO1 and DOI2 has been shown in parrotfish, with higher expression levels in ovaries than testes, suggesting that ovaries may require more bioactive THs than testes (234). Whereas there is no evidence for a role of DIO1 in the gonads, DIO2 has been implicated in the regulation of gonad maturation and gametogenesis. In zebrafish, DIO2 deficiency results in delayed sexual maturity and reduced gametogenesis and spawning in both males and females (240). Conversely, high DIO2 activity/expression in gonads [e.g., female tilapia (216); male rainbow trout, (239)], may ensure appropriate levels of T_3_ needed for gametogenesis. In the sapphire devil, transcript levels of ovary DIO3 increase as vitellogenesis progresses, suggesting that high DIO3 expression might prevent excess TH buildup (238).

#### Thyroid Hormone Receptors

TRs are expressed in gonads of teleosts such as goldfish (103, 236), striped parrotfish (234), Korean rockfish (241), black porgy (*Acanthopagrus schlegelii*) (242), and fathead minnow (243), and their expressions appear to be gender-dependent and species-specific. The expressions of TRα and TRβ are higher in ovary than in testis in mature Korean rockfish (241), mature goldfish (103), and developing fathead minnow (243), but higher in testis than the ovary in striped parrotfish (234).

In fish that change sex as part of their life-history strategy, TR subtypes display expression changes in regard to gender. In protandrous (sex change from male to female) black porgy, TRα mRNA expression is low in immature testis and increases at maturation. During sex change, TRα expression decreases then subsequently increases during ovary development and maturation and TRβ expression is highest in mature ovary after sex change than in any other gonadal or sex stage (242). These results suggest that TRα is critical for both testis and ovary development, and TRβ might only be required in the ovary of this species, similar to fathead minnow (243). The significance of this differential expression is yet to be uncovered, but most likely important in cell-specific proliferation and differentiation in gonads, albeit, dependent on sex.

#### Thyrotropin Receptors

Thyrotropin receptor (TSHR) expression has been detected in gonads of several species, including European sea bass (244), walking catfish (*Clarias batrachus*) (245), channel catfish (246), striped bass (*Morone saxatilis*) (247), Biwa trout (*Oncorhynchus rhodurus*) (248), and sunrise sculpin (*Pseudobennius cottoides*) (248).

TSHR expression levels increase during ovarian and testicular maturation in European sea bass (244), channel catfish (246) and striped bass (247), and peak during spermatogenesis in sunrise sculpin (248), suggesting a direct role of TSH and TSHR in gametogenesis. In walking catfish, GnRH treatment increases TSHR mRNA expression in gonads, suggesting a positive correlation between TH levels and reproduction (245).

### Thyroid and Hypothalamus-Pituitary-Gonadal Axes

In fish, as in mammals, the thyroid influences the HPG axis in a gender-, development-, and species-specific manner. The effects of the thyroid axis on reproductive processes of fish occur *via* actions at all levels of the HPG axis, i.e., the hypothalamus, pituitary, and gonads.

In the hypothalamus, the effects of THs on GnRH appear to depend on the species and the reproductive-stage considered, as well as the specific population of GnRH neurons. In male mature recrudescent (active gametogenesis) air-breathing catfish (*Clarias gariepinus*), thiourea-induced TH depletion reduces the number of hypothalamic GnRH immunoreactive neuronal cells and fibers (249). In immature male Nile tilapia, T_3_ treatment suppresses terminal nerve GnRH mRNA, but does not significantly affect preoptic or midbrain GnRH mRNA levels or the number of hypothalamic GnRH neurons (250), suggesting central-specific TH action dependent on reproductive stage.

Studies have shown that THs may act at the pituitary level to inhibit gonadotropin secretion. Hypothyroid conditions decrease pituitary LH immunoreactivity and LH circulating levels in male recrudescent air-breathing catfish (249), and, in recrudescent goldfish, administration of T_3_ decreases pituitary LH mRNA expression in males (251) and attenuates GnRH-induced LH secretion in females (252).

Gonadal steroidogenesis occurs in Leydig cells of testes and thecal and granulosa cells of ovaries, and starts with the transport of cholesterol into the mitochondria mediated by steroidogenic acute regulatory protein (StAR), where it is converted into pregnenolone, which is sequentially converted into active steroids such as progesterone (P), 17α-hydroxy-20β-dihydroprogesterone (DHP), the androgens testosterone (T) and 11-ketotestosterone (11-KT, the predominant androgen in fish), and estradiol-17β (E_2_) by several steroidogenic enzymes (253). In male vertebrates, Sertoli and Leydig cells are responsible for spermatogenesis and androgen biosynthesis, respectively, whereas oogenesis is stimulated by ovarian estrogen and progestins in females (254).

There is evidence in fish that THs increase spermatogenesis and androgen secretion in males and estrogen and progestin secretion in females. In zebrafish testis, T_3_ stimulates spermatogenesis by increasing the division of spermatogonia and Sertoli cells (255, 256), increasing the production of IGF-III (insulin-like growth factor-III, a stimulatory growth factor of spermatogenesis) by Sertoli cells, and enhancing the gonadotropin-induced synthesis and release of androgens by Leydig cells (257). In male goldfish, treatment with T_3_ decreases expression of CYP19 (aromatase, which converts androgens into estrogens) thus increasing the androgen to estrogen (A:E) ratio (251), and inhibiting T_3_ synthesis with monocrotophos (organophosphate pesticide) increases CYP19 expression and reduces the A:E ratio (258). In contrast, in cultured adult zebrafish testis, T_3_ does not affect the release of 11-KT, or AR and CYP19 mRNA expressions (255), and in juvenile common carp, treatment with T_4_ has no effect on testis diameter or number of spermatogonia (259). In mid to late recrudescent male goldfish, T_3_ decreases circulating E_2_ levels and expression levels of testis estrogen receptor subtypes (ERα, ERβ1, and ERβ2) during mid-recrudescence (251), but has no effect in late or regressed gonads (51). This suggests that THs are essential for spermatogenesis in males but are reproductive stage-specific and seem to have the greatest effect in periods of active spermatogenesis.

In mid-recrudescent female goldfish, *in vivo* T_3_ treatment decreases the expressions of estrogen receptors (ERα and ERβ1) and CYP19 in ovary (251), and in recrudescent female air-breathing catfish, T_4_ treatment decreases CYP19 immunoreactivity and E_2_ levels in ovary (260), while thiourea-induced TH depletion increase ovarian expression of CYP19 (261). In oocytes of pre-spawning climbing perch, *in vitro* T_3_ treatment increases progesterone release (262) and 3β-hydroxysteroid dehydrogenase (3β-HSD, which converts pregnenolone to progesterone) activity (263), and enhances gonadotropin-induced E_2_ secretion in ovarian follicles from spawning rainbow trout (264).Therefore, similar to male testes, the actions of TH in ovaries appear more pronounced during active periods of gametogenesis. It has been suggested that in seasonal species such as goldfish, THs might inhibit oogenesis/vitellogenesis during non-spawning season, allowing fish to allocate their energy to somatic growth (251, 265).

Very few studies have been performed in elasmobranchs. In the oviparous female dogfish, thyroidectomy impairs ovarian follicular development (224). Both male and female spiny dogfish show correlations between gonad follicle and thyroid growth, with female follicular cell height showing a positive relationship to thyroid weight (228).

While THs affect reproductive tissues, the thyroid axis is also regulated by reproductive hormones. In fish, treatment with E_2_ appears to have inhibitory effects on TH levels, as seen by E_2_ induced decrease in thyroid epithelial cell height and thyroid activity [e.g., European eel (266) and rainbow trout (267)], decreases in plasma TH levels (usually T_3_) [e.g., European eel (266), Atlantic salmon (268) and southern hemisphere lamprey (*Geotria austrails*) (269)], decreases in hepatic T_3_ production [e.g., trout (264, 270) and masu salmon (271)], increases in TSH [e.g., rainbow trout (270) and masu salmon (271)], and decrease in gonad TRα expression in male and female fathead minnow (272). Like estrogens, androgens might also affect the thyroid axis in fish (273). Androgens have been shown to enhance thyroidal function in most teleosts examined [e.g., striped catfish (*Mystus vittatus*) (274); rainbow trout (275); masu salmon (276); coho salmon (277), striped catfish (274)]. In Japanese medaka (*Oryzias latipes*) (278) and coho salmon smolt (277), 11-KT (medaka), and 17α-methyltestosterone (MT, coho) administration in larval males causes thyroid follicle hypertrophy and enhances 5’-MDA activity (279). However, MT treatment induces a dose-dependent decrease in plasma T_4_ and inhibits the smoltifying effects of T_4_ in masu salmon (276).

## Role of Thyroid Hormones in Osmoregulation

In mammals, the kidney is the major osmoregulatory organ, and THs influence renal development, kidney hemodynamics, glomerular filtration rate and ion and water homeostasis (280) and thyroid dysfunction affects renal function (280).

In fish, osmoregulation is accomplished by the kidneys and GIT, but mainly by gills (*via* chloride cells) in teleosts and rectal gland in elasmobranchs (281). Compared to the outside water, the internal environment of marine fish is hypoosmotic, while that of a FW fish is hyperosmotic. Most species live in relatively constant habitats and can only survive within a narrow range of salinities (stenohaline). However, other species are able to adapt to a wide range of salinities (euryhaline) and some undergo drastic osmotic changes as they migrate [from SW to FW (anadromy) or from FW to SW (catadromy)] (282).

Several hormones control osmoregulation in fish. In euryhaline fish, cortisol (a glucocorticoid secreted by kidney) is considered the main SW adapting hormone whereas prolactin (PRL, which promotes ion uptake and inhibits ion secretion) is viewed as a FW adapting hormone; GH and IGF-I have also been implicated in the control of SW adaptation (283, 284). The thyroid axis has been shown to regulate osmoregulatory changes in fish, most likely through interactions with cortisol/GH and PRL (283, 284).

### Salinity Tolerance in Salmonids

Several studies have examined the role of the thyroid axis in determining tolerance to changing salinities in salmonids. Salinity tolerance (capacity to withstand SW) increases after TH treatment in FW coho salmon (285, 286), Atlantic salmon (287, 288), pink (*Oncorhynchus gorbuscha*) and sockeye salmon (289), and sockeye salmon transferred from FW to SW have increased gill TRα, TRβ1, and TRβ2 mRNA and increased TH levels (290). In Atlantic salmon, T_3_ increases the binding affinity of cortisol to gill cortisol receptors, an effect synergistic when co-injected with GH (291)—indicative of increased SW tolerance. In amago salmon (*Oncorhynchus rhodurus*), T_4_ treatment potentiates the action of GH on gill Na^+^/K^+^-ATPase (NKA, major ion pump) (292), while there is a synergistic effect in gill NKA activity in Atlantic salmon (291) and rainbow trout (293) when co-injected with T_3_ and GH.

Atlantic salmon injected with PRL limits cortisol receptor binding affinity and decreases NKA activity, reducing SW tolerance. In coho salmon, PRL alone has no effect on plasma T_3_ levels and decreases plasma T_4_ levels, and when PRL is co-injected with TSH it prolongs the TSH-induced elevation of TH levels (294). In brook trout (*Salvelinus fontinalis*) co-injections of TSH and PRL increase plasma T_3_ levels, hepatic T_3_ content and 5’-MDA rates compared with TSH-treated animals (295), suggesting an interaction between TSH and PRL.

### Evidence in Other Euryhaline Fish

THs have also been shown to affect the osmoregulatory capabilities of other euryhaline species. In Mozambique tilapia, TH injections increase gill NKA activity (296), potentiate the action of cortisol on gill NKA activity (297) and increases chloride cell size (a function of ionoregulatory ability) (296).

In summer flounder (*Paralichthys dentatus*), which move from high to low salinity ocean water during metamorphosis, SW tolerance increases after TH treatment in individuals undergoing metamorphosis, suggesting that, similar to anadromous salmon, THs regulate the development of osmoregulatory mechanisms necessary for the transition to FW to SW (298). In gilthead sea bream (*Sparus aurata*), exposure to low salinity increases T_4_ levels and decreases gill DIO1 activity (299), while high salinity decreases T_4_ levels and increases pituitary TSHβ and gill NKA activity (300). However, in grass carp (*Ctenophayngodon idella*), an increased salinity decreases T_3_ and TSH levels, and increases T_4_ serum levels (301).

Marine and euryhaline elasmobranchs in SW regulate urea and other body fluid solutes [trimethylamine oxide (TMAO), Na^+^, Cl^−^] such that they remain iso- or slightly hyperosmotic to their environment (302). While little information is available, it seems that the thyroid axis may contribute to elasmobranch osmoregulation. In Atlantic stingray, plasma urea levels and osmotic concentration increase following thyroidectomy and decrease after T_4_ replacement therapy, possibly due to the regulation of urea efflux or metabolism (303). In dogfish, 5’-MDA liver activity increases in the presence of TMAO (protein stabilizer that counteracts urea buildup) and TMAO + urea (136), suggesting a role of THs in urea metabolism, as seen in goldfish, for which T_4_ increases ammonia production and excretion (304, 305).

## Feeding and Nutrient Homeostasis

The nutritional energy provided by food intake is essential for activity, growth, and maintenance of bodily functions. In fish (306) as in mammals (307), food intake is mainly regulated by brain feeding centers controlled by central and peripheral endocrine signals, which either stimulate [orexigenic peptides, such as orexin, agouti-related protein (AgRP), and neuropeptide Y (NPY)] or inhibit [anorexigenic signals, such as cocaine- and amphetamine-regulated transcript (CART) and α-melanocyte-stimulating hormone (α-MSH) derived from POMC] feeding behavior. Feeding centers receive information about nutritional status from the periphery [*e.g.*, gastrointestinal tract (GIT)] either *via* the general circulation or the brainstem/vagal complex. These peripheral signals include ghrelin, cholecystokinin (CCK), peptide YY (PYY), and leptin. Usually, when food intake is restricted, the expression of orexigenic hormones increases while that of anorexigenic hormones decreases (306, 308).

### Role of the Thyroid Axis in Feeding/Food Intake

In mammals, the thyroid axis regulates food intake, body weight (309) and metabolic/nutrient homeostasis (310). The thyroid axis can influence feeding *via* the actions of TRH and THs in the brain, THs in the periphery, and also be influenced by endocrine appetite-regulating signals (e.g., NPY, leptin).

In rodents, central administration of TRH or TSH decreases food intake (311, 312) whereas TH injections increase feeding (313, 314). Conversely, food deprivation decreases hypothalamic TRH and pituitary TSHβ mRNA expression, and peripheral T_3_ serum levels (315), while refeeding increases hypothalamic TRH mRNA expression, increases plasma TSH, and normalizes circulating T_3_ levels (316).

Interactions between the thyroid axis and appetite-regulating signals have been shown in mammals. In rats, although TRH neurons contain NPY receptors (317), TRH does not stimulate NPY neurons (318), but goats injected with NPY show a dose-dependent increase in TH levels (319). TRH neurons excite orexin neurons (318) and orexin has been reported to either increase (320) or decrease (321) hypothalamic TRH levels. Interestingly, some hypothalamic TRH neurons co-secrete CART but the nature of this interaction is unclear (322). It has been suggested that the anorexigenic actions of TRH are mediated in part by the inhibition of melanin-concentrating hormone (MCH, an orexigenic neuropeptide) (318), while the orexigenic effect of THs might occur *via* decreases in the expression of anorexigenic factors such as POMC, CART, and MC4R (melanocortin 4 receptor, activated by *α*-MSH and AgRP to reduce food intake) (310, 323, 324), and increases in the expression of appetite stimulators such as NPY (325). Leptin (a adipose satiety signal) increases TRH expression directly by binding to its receptors at TRH neurons (326), or indirectly *via* decreases in AgRP and NPY and increases in *α*-MSH (which innervate TRH neurons) (309, 327). There is no clear evidence of a correlation between THs and leptin expression and circulating levels (328, 329).

In fish, interactions between feeding and thyroid status have been shown in several species. In green sunfish (*Lepomis cyanellu*s), high thyroid activity correlates with increased food intake (330), whereas in Amur sturgeon (*Acipenser schrenckii*), low serum TH levels correlate to low feeding rates (331). In climbing perch, exposure to thiourea (TPO inhibitor) decreases food consumption (332). Reduced food ration in green sunfish (330) and long-term starvation in rainbow trout (333) decreases the sensitivity of thyroid tissues to TSH, resulting in a decrease in TH levels. In winter flounder, hypothalamic TRH expression increases during fasting (334) but decreases in common carp (335), and in goldfish, TRH injections increase food intake (336).

Little is known about interactions between the thyroid axis and appetite regulators in fish. In goldfish, TRH injections increase the brain expression of orexin, orexin receptor, and CART (336). In bighead carp pituitaries, leptin increases TSHα and TSHβ expression (42), and in grass carp, leptin and ObRb expression levels increase in hepatocytes incubated with low doses of T_3_ (although high doses inhibit expression) (337). In fasted burbot, plasma T_4_ and TSH correlate with increased plasma leptin levels (180).

All together, these results suggest that in fish, the thyroid axis plays a role in regulating appetite, and responds to changes in feeding status.

### Thyroid Hormones, Nutrient Synthesis, and Metabolism

Nutrients and how efficiently they are metabolized have been shown to influence and be influenced by the thyroid axis. In mammals, hyperthyroidism is associated with high metabolism—increased fat breakdown, weight loss, increased liver cholesterol synthesis and clearance, and low serum cholesterol—while the opposite occurs in hypothyroidism (338). For example, in rats, T_3_ increases caloric intake and leads to increased lipolysis (by fatty acid β-oxidation) (339), while hypothyroid female rats have reduced hepatic mRNA expressions associated with cholesterol uptake and lipid oxidation (340). Conversely, the quality of nutrients influences the thyroid axis and TH production. Rats fed fish oil diets have higher liver TR expression and increased thyroid signaling associated with lipid metabolism than rats fed soybean oil diets (341), and rats fed diets supplemented with *Yucca schidigera* (which contains saponins that decrease GIT nutrient absorption), have lower THs levels than control animals (342).

In fish, THs influence nutrient metabolism of lipids, proteins and carbohydrates (343) in a species-specific manner. T_4_ treatment promotes lipolysis, stimulates lipid mobilization, and decreases lipid stores (e.g., as seen by decreased total lipids and increased lipolytic enzyme activity) in coho salmon (344), and increases lipid efficiency, plasma cholesterol and triglyceride levels in Sterlet sturgeon (345). Body protein content decreases in European eel (glass stage) treated with THs (346), and walking catfish exposed to thiourea (347). THs also affect glucose and related carbohydrate metabolism pathways. Following TH treatment, plasma glucose levels increase in red sea bream (348), gilthead sea bream (349), and European eel (346), but decrease in rainbow trout (350). TH treatment increases liver gluconeogenic pathways in gilthead sea bream *in vivo* (349), and expression of transcripts associated with glycolytic pathways [i.e., glucokinase (GK), glucose-6-phosphatase (G6Pase), glycogen synthase (GS), and glycogen phosphorylase (GP)] in silver sea bream hepatocytes *in vitro* (351). However, RNA-seq analysis conducted in liver of tilapia treated with T_3_ shows a down-regulation of several pathways related to carbohydrate metabolism (i.e., amino sugars synthesis, galactose and mannose metabolism, tricarboxylic acid cycle) (116).

The quality of the food (i.e., protein, carbohydrate, or lipid content) also influences the thyroid axis in fish. For example, low protein diets reduce plasma T_4_ levels and/or 5’-MDA activity in rainbow trout (352) and brook trout (353). Similarly, in Japanese flounder, fish meal-fed fish have higher levels of T_3_ than fish fed with fish protein concentrate (FPC) or soy protein concentrate (SPC) (353). Rainbow trout fed a diet with low carbohydrates have low 5’-MDA activity compared to fish fed a carbohydrate-rich diet (354). Under a diet with low salmon oil content, rainbow trout have reduced plasma T_4_ and increased plasma T_3_ levels, while a high salmon oil diet leads to high plasma T_4_ and low T_3_ (354).

## Relevance of the Thyroid Axis in Aquaculture

The basic premise to aquaculture systems is to maximize growth at a minimum cost, producing an aesthetic product with high nutritional value (355). The bottlenecks in aquaculture are often the survival of larval and juvenile stages, and successful spawning. Manipulations or disruptions of the thyroid axis could potentially have positive (e.g., increased developmental and reproductive success, hatching, and growth rates) or negative (e.g., skeletal deformations, depressed food intake) effects in the aquaculture industry.

### Thyroid Hormones Could Be Used to Enhance Early Survival and Development in Fish

THs are important in the development and growth of fish, particularly during early life stages. In aquaculture settings, high mortality rates are seen in early life stages and several species develop skeletal deformities or abnormal pigmentations which might compromise the aspect of the fish and render it improper for sale [e.g., Atlantic salmon (356); Atlantic cod (357); flatfish (358)].

Many studies have reported positive effects of TH treatment in newly fertilized eggs and larvae to enhance hatching, post-embryonic growth and larval survival. For example, immersion in T_4_ reduces the hatching period, the number of physical deformities, and mortality rate in Asian stinging catfish (*Heteropneustes fossilis*) eggs, (359), and induces faster development (i.e., gut formation, swim bladder development, yolk absorption) in freshwater carp (*Catla catla*) larvae (360). Similar positive effects have been shown in Pacific threadfin (*Polydactylus sexfilis*) (361), spotted gar (128), rainbow trout (362), milkfish (*Chanos chanos*) (363), grouper (202), and chum salmon (364), as well as a number of South American fish [e.g., piracanjuba (131); matrinxã (132)*;* dourado (*Salminus maxillosus*) (365)].

However, negative effects of THs have also been reported. T_4_ immersion results in reduced hatching, growth rate, and yolk content in alligator gar (128), decreased pigmentation in Atlantic salmon (366), major abnormalities in Nile tilapia [i.e., abnormal shaped pectoral fins, lordosis, and scoliosis (spinal curvature)] (367) and albinism in Japanese flounder—possibly *via* inhibition of pigment production or impairment of melanophore development due to precocious metamorphosis (368).

Overall, these studies suggest that the effects of TH on eggs and larvae might be dose- and species-dependent.

### Thyroid Hormones Can Control and Optimize the Time of Salmonid Smoltification

As there are individual variations in growth rates in fish, THs (which are involved in stimulating both growth and smoltification) have been used to accelerate growth and promote the achievement of SW tolerance in several salmonids (369). TH treatments could also be useful in inducing promote out-of-season growth and smoltification.

Smoltification is controlled by environmental cues (mainly photoperiod and temperature), which induce changes in the thyroid axis (370–372) and only occurs when a threshold weight has been reached (373). In aquaculture, the period following the transfer of fish from FW to SW is critical, as the performance (including optimal growth rates) of the fish after transfer depends upon a successful Parr-Smolt transformation (374).

A well-timed TH induction of smoltification may be advantageous in species which are released and recaptured [e.g., kokanee salmon (*Oncorhynchus nerka*) (375)] to ensure the return of adult fish to release sites, as fish with the highest whole body T_4_ content display increased odor attractions and more accurate homing behavior compared to fish with low T_4_ levels (376). In Atlantic salmon smolts following transfer to SW, there is a transient suppression of appetite and growth (for up to 30 days) (374, 377), and THs treatment at the right time and the right dose during the parr phase might lessen this inhibition. However, T_4_ administration in late Atlantic salmon parr depressed olfactory bulb response to L-alanine (nasal stimulant in salmon) and inhibited 5’-MDA, so timing of induction is critical (378).

### Thyroid Hormones Could Enhance Reproduction

THs may potentially be used to enhance reproduction in some aquaculture species by enhancing offspring survival and market value [e.g., increase quality of eggs for sturgeon caviar production (379)]. Higher embryonic/larval survival rates and hatching rates have been shown in fertilized eggs treated with THs [e.g., Pacific threadfin (361); Sterlet sturgeon (130)] or following maternal TH injections [e.g., greater amberjack (*Seriola dumerili*), Japanese whitling (*Sillago japonica*), red spotted grouper (*Epinephelus akaara*), red sea bream, and Japanese parrotfish (*Oplegnathus fasciatus*) (380, 381); striped bass (382)]. In Medaka, administration of T_3_ prior to spawning increases E2 production and oocyte growth, showing that T_3_ administration can enhance final oocyte maturation (383).

The use of THs to enhance reproduction has been successfully used in large scale aquaculture production of some species [e.g., goldstriped amberjack (*Seriola lalandi*) (380); Korean rockfish (137)]. In goldstriped amberjack, maternal injections of T_3_ reduce mortality during early development and growth, and larval survival increased from less than 1.0% when seed production began in 1985, to 7.3% by 1994 following implementation of T_3_ injections (380).

### Thyroid Disruption by Anthropogenic Actions as a Threat to Aquaculture

#### Pollutants

Thyroid disruption by exposure to environmental toxicants such as metals [e.g., cadmium (384)], pesticides [e.g., organophosphorous pesticides (385)], and pollutants [e.g., polychlorinated biphenyls, PCBs (386)] could result in increased larval mortality and developmental deficiencies (387) depending on the aquaculture system and species.

With increasing anthropogenic and industrial activities, heavy metals can become soluble and accumulate to toxic levels, and potentially affect the thyroid axis (388). Cadmium decreases TH levels in rainbow trout (389), while chromium exposure reduces TH levels in European eel (390), and induces thyroid follicle hypertrophy and increases in serum TH levels in spotted snakehead (*Channa punctatus*) (391). Exposure to mercury decreases circulating TH levels in spotted snakehead (392) and increases the T_4_:T_3_ ratio—suggesting an inhibition of 5’-MDA activity—in yellowfin sea bream (*Acanthopagrus latus*) (393).

Organophosphorus pesticides (OPs) can inhibit growth and development of fish. Dimethoate decreases serum TH levels and increases TSH levels in roho labeo (*Labeo rohita*) (394), chlorpyrifos decrease serum TH and TSH levels in Asian stinging catfish (395), and decreases in TH levels inhibits development of sensory organs (eyes, olfactory organ, and lateral line) and decreases survival rates in surgeonfish (396). In goldfish, monocrotophos decrease TH levels, and up-regulate pituitary TSHβ and hepatic DIO1 and DIO3 expressions (397). In Senegalese sole, exposure to malathion affects growth patterns (eye migration, skeletal disorders), reduces thyroid follicle size, and induces decreased thyroid signaling (as seen by low TRβ mRNA levels) (398).

PCB exposure induces higher rates of thyroid metabolism (i.e., deiodination, glucuronidation, and sulfatation) and lower TH levels in European sea bass (399), coho salmon (400), and rainbow trout (401), but not in European flounder (*Platichthys flesus*) (402).

Therefore, while some mechanisms of interaction between environmental toxicants and the thyroid axis are unknown, toxicants can have negative effects on thyroid economy of fish, and could potentially affect growth and production of aquaculture species.

#### Climate Changes

Climate change brings about changes in the aquatic environment, such as increases in temperature and acidification, which deeply affect fish physiology (403) and aquaculture practices (404), and might have potential effects on the thyroid axis.

Warmer temperatures have been shown to decrease the sensitivity of fish to THs in zebrafish (118, 405) and mosquito fish (*Gambusia holbrooki*) (406), and in surgeonfish, a 3°C increase in temperature induces lower TH levels and a disrupted development of sensory organ, an effect that can be reversed by treating the fish with THs (396). In addition, thermally challenged fish may produce less viable gametes, with fitness implications that could affect species at the population level (407). In Japanese Medaka, high temperatures decrease the number of spawned eggs, an effect amplified by a reduction in TH levels (by sodium perchlorate exposure) (408). Similarly, seasonal spawners such as goldfish exhibit high TH levels post-spawning in the summer (when water temperatures are the highest) as a way to inhibit pituitary LH and gonadal aromatase (265). While these temperature-mediated effects have not held true for all fish species [e.g., Atlantic cod (84, 409)], an earlier than normal increase in water temperatures as a result of climate change, might disrupt thyroid cycles and inhibit reproductive capabilities in some fish.

The thyroid axis is also sensitive to ambient acidity. For example, exposure to acid water increases T_4_ plasma levels in the climbing perch (*Anabas testudineus*) (410) and brown trout (*Salmo trutta*) (411), and a decrease in T_3_ levels in Atlantic Salmon (412).

Changes associated with climate may differentially affect specific life-history stages of fish (e.g., species that undergo substantial metamorphic events), which may result in plastic responses that lead to deficiencies later in life. These abiotic changes are poorly understood in the context of the thyroid axis and fish, but require attention for future climate scenarios and aquaculture practices.

## Summary and Conclusion

**Figure 1:**
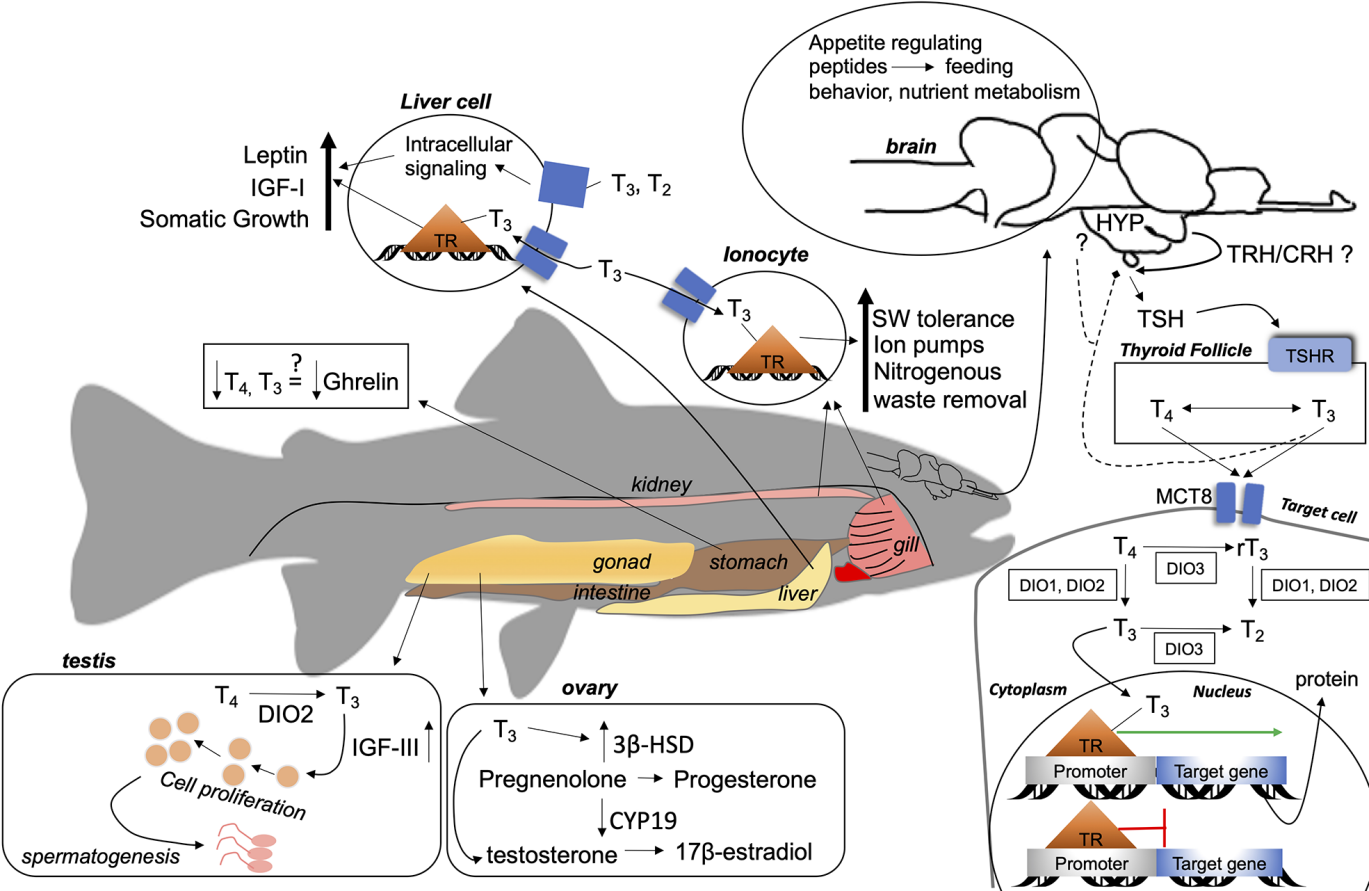
A summary of the general actions of thyroid hormones (THs) in fish. A thyrotropin (TSH)-releasing factor [thyrotropin-releasing hormone (TRH)/corticotropin-releasing hormone (CRH)] stimulates the anterior pituitary to release TSH, which binds to TSHR on the membrane of thyroid follicles. Intracellular processes produce T_4_ and T_3_ that enter the circulation to target cells (solid line) or feedback (dashed line) to the hypothalamus–pituitary axis. THs enter target cells through membrane transporters (e.g., MCT8), where bioactivation of T_4_ to T_3_ occurs through DIO1 and DIO2, or further metabolization to rT_3_ or T_2_ through DIO1, DIO2, or DIO3. THs enter the target cells nucleus from the cytoplasm and bind to TRs located on promoter regions of a thyroid hormone response element (TRE). When T_3_ is bound, gene transcription occurs (green arrow), otherwise transcription is repressed (red line). THs may act on various tissues in fish, as shown by general mechanisms in central and peripheral tissues. Question marks indicate evidence of effects of THs, but no known mechanism of action by THs in fish. Arrows that point up indicate that THs increase activity, production, or synthesis. Down arrows indicate repression or reduction of synthesis/production. *HYP*, hypothalamus; *TRH*, thyrotropin-releasing hormone; *CRH*, corticotropin-releasing hormone; *TSH*, thyrotropin, *TSHR*, thyrotropin receptor; *MCT8*, monocarboxylase transporter 8; *T_4_* thyroxine, *T_3_* triiodothyronine; *rT_3_*, reverse triiodothyronine; *T_2_*, diiodothyronine; *DIO1*, deiodinase I; *DIO2*, deiodinase II; *DIO3*, deiodinase III; *TR*, thyroid receptor; *IGF-I*, insulin-like growth factor I; *IGF-III*, insulin-like growth factor III; *3β-HSD*, 3β-hydroxysteroid dehydrogenase; *CYP19*, aromatase.

Thyroid hormones have diverse effects and play an important role in the maintenance of a normal physiological state in vertebrates. While similarities exist between fish and other vertebrates exist, fish thyroidal systems present unique features (see **Table 1**, **Figure 1**) and functions owing to the diversity in fish anatomies, habitats, and life cycles.

**Table 1:** Example effects of the thyroid axis on various physiological processes in fish.

Process		Effects
Egg/larval survival	**+**	Thyroxine increases egg viability, hatchability, and survival [e.g., common carp (363)].
Egg/larval/juvenile development	**+**	TH immersion or injection increases pigmentation, hatching, growth rate, larval metabolic capacity [e.g., Sterlet sturgeon (125, 130); zebrafish (133); goldfish (134)].
	**−**	Hyperthyroidism leads to arrested development of skeletal structures [e.g., zebrafish (149)].
Juvenile/adult development	**+**	T_4_ induces opsin switch in juvenile coho salmon and rainbow trout (183). T_4_ promotes intestinal and swim bladder development in freshwater carp larvae (360). T_3_ and T_2_ promote growth in tilapia (119).
Metamorphosis/smoltification	**+**	THs increase olfactory bulb proliferation, body silvering, and downstream migration in salmon (196, 197).
	**−**	Metamorphosis is blocked by THs in sea lamprey (191).
Reproduction	**+**	T_3_ stimulates spermatogenesis in zebrafish by increasing IGF-III (255, 256). T_3_ increases progesterone release in female climbing perch (262).
	**−**	T_3_ treatment suppresses terminal nerve GnRH expression in Nile tilapia (250) and administration of T_3_ in male goldfish decreases pituitary LH mRNA expression (251).
Osmoregulation	**+**	T_3_ injections increase gill ion pump activity in Mozambique tilapia (296) and T_4_ immersion increases salinity tolerance in summer flounder (298).
Feeding/food conversion	**+**	TRH injections increase food intake in goldfish (336). T_4_ increases food, protein, and lipid efficiency in sterlet sturgeon (345).
	**−**	T_3_ decreases body protein in European eel (346) and decreases plasma glucose in rainbow trout (350).

A (+) denotes the thyroid axis enhancing the physiological process while a (−) denotes a suppression or impairment.

The follicular structure of the thyroid is conserved in vertebrates, but most fish have diffuse glands making it more difficult to study. The mechanisms by which fish synthesize and metabolize THs is similar to those in mammals (i.e., THs requires thyroglobulin, iodine, and TPO, and DIOs are needed to activate/inactivate THs), but fish might have different isoforms of enzymes which have different properties/actions/locations (e.g., DIO1 is insensitive to PTU and DIOs are located in various tissues), suggesting diverse TH metabolisms.

Evidence suggests that TRH may not be the major TSH-releasing factor at the pituitary in fish, but rather be responsible for the secretion of GH, PRL, and ACTH, which in turn might affect TSH. THs appear to exert an inhibitory feedback action on TSH, but there is no clear evidence for TRH. More advanced molecular techniques (e.g., RNA-sequencing) and *in vivo* studies may help to shed light on the true nature and interactions of TRH in fish.

Existing literature has highlighted the actions of TH in fish *via* genomic (binding to species specific isoforms of TRs) mechanisms. However, the non-genomic mechanisms by which THs act are poorly understood, as these processes can overlap with genomic actions. As in all vertebrates, T_3_ is the main biologically active form of TH, but metabolized THs (e.g., T_2_ and Tetrac) previously deemed inactive, are proving to have a role in regulating metabolism (413).

In fish, THs regulate many aspects of reproduction, including gonad maturation, steroidogenesis, and sexual behavior, and can affect the time of spawning, quality of eggs, and fertilization rates and development of eggs/larvae. There are also deep complex interactions between the thyroid axis and growth (e.g., GH, IGF-1) and feeding/appetite (e.g., NPY, POMC) regulators, however, a good knowledge of these interactions is still lacking. A better understanding of the control of THs on reproduction, growth, and development, and feeding might provide invaluable insights in aquaculture species/practices and may especially be important to maximize growth while reducing production costs in the ever-growing aquaculture industry.

Any alteration of the thyroid axis by environmental anthropogenic pollutants (effluents containing thyroid disrupting compounds) could have serious physiological and ecological consequences. Understanding specific mechanisms of action of these pollutants might help to substantiate their potential long term affects, and help fisheries managers regulate wild populations under threat from these compounds.

Finally, climate change is an additional stress to aquatic ecosystems, affecting both water temperature and shifting carbon dioxide concentrations through direct and indirect effects. Owing to the aquatic habitat of fish, the thyroid axis shows trends in seasonality (414), and is affected by external factors such as temperature, salinity, and pH (118), begging the question on how climate change might alter thyroid signaling.

## Author Contributions

CKD and HV both designed, wrote, and approved the final version of the manuscript.

## Funding

The authors acknowledge funding from the Natural Sciences and Engineering Research Council (NSERC) Discovery Grant, 261414-03 (HV).

## Conflict of Interest

The authors declare that the research was conducted in the absence of any commercial or financial relationships that could be construed as a potential conflict of interest.
